# Comparison of Human-Like H1 (*δ*-Cluster) Influenza A Viruses in the Swine Host

**DOI:** 10.1155/2012/329029

**Published:** 2012-06-03

**Authors:** Janice R. Ciacci Zanella, Amy L. Vincent, Eraldo L. Zanella, Alessio Lorusso, Crystal L. Loving, Susan L. Brockmeier, Phillip C. Gauger, Bruce H. Janke, Marie R. Gramer

**Affiliations:** ^1^Virus and Prion Diseases Research Unit, National Animal Disease Center, Agricultural Research Service, USDA, Ames, IA 50010, USA; ^2^Labex-USA, Brazilian Agriculture Research Corporation (EMBRAPA), 70770-901 Brasília, DF, Brazil; ^3^Embrapa Swine and Poultry Research Center, 89700-000 Concórdia, SC, Brazil; ^4^College of Agronomy and Veterinary Medicine, University of Passo Fundo, 99052-900 Passo Fundo, RS, Brazil; ^5^Department of Veterinary Diagnostic and Production Animal Medicine, College of Veterinary Medicine, Iowa State University, Ames, IA 50011, USA; ^6^University of Minnesota Veterinary Diagnostic Laboratory, Saint Paul, MN 55108-1098, USA

## Abstract

Influenza A viruses cause acute respiratory disease in swine. Viruses with H1 hemagglutinin genes from the human seasonal lineage (*δ*-cluster) have been isolated from North American swine since 2003. The objective of this work was to study the pathogenesis and transmission of *δ*-cluster H1 influenza viruses in swine, comparing three isolates from different phylogenetic subclusters, geographic locations, and years of isolation. Two isolates from the *δ*2 subcluster, A/sw/MN/07002083/07 H1N1 (MN07) and A/sw/IL/00685/05 H1N1 (IL05), and A/sw/TX/01976/08 H1N2 (TX08) from the *δ*1 sub-cluster were evaluated. All isolates caused disease and were transmitted to contact pigs. Respiratory disease was apparent in pigs infected with MN07 and IL05 viruses; however, clinical signs and lung lesions were reduced in severity as compared to TX08. On day 5 following infection MN07-infected pigs had lower virus titers than the TX08 pigs, suggesting that although this H1N1 was successfully transmitted, it may not replicate as efficiently in the upper or lower respiratory tract. MN07 and IL05 H1N1 induced higher serum antibody titers than TX08. Greater serological cross-reactivity was observed for viruses from the same HA phylogenetic sub-cluster; however, antigenic differences between the sub-clusters may have implications for disease control strategies for pigs.

## 1. Introduction

Influenza A viruses are important infectious agents for humans, avian species, and many mammalian species, including swine. In swine, influenza virus causes an acute infection characterized by high morbidity and very low mortality rates [1]. Influenza viruses of the family *Orthomyxoviridae* have negative-sense single-stranded eight-segmented genome encoding for up to twelve structural and accessory proteins [2]. The triple reassortant internal gene constellation (TRIG) is the common backbone of the swine influenza viruses currently circulating in North America (for a review, see Vincent et al., 2008 [3]). Within the TRIG viruses, a dominant circulating genotype carries the HA and NA encoding genes of the human seasonal viruses of the H1 lineage (hu-like), identified from pigs in American and Canadian herds [4]. The HA genes of these viruses form the *δ*-cluster in phylogenetic analyses of HA genes from North American influenza A viruses of swine. Contemporary *δ*-cluster HA genes can be further divided into two sub-clusters, *δ*1 and *δ*2 [5].

Phylogenetically, the HA sequences of American and Canadian *δ*-cluster influenza A viruses appeared to have been derived from at least two independent human-to-pig transmission events [3, 6]. The NA genes were also atypical of classical H1N1 swine viruses, forming a cluster of N1 separate from the swine lineage N1 either and more similar to human H1N1 from 2002-2003 or similar to human-like N2 from the same lineage as the triple reassortant H3N2 swine viruses that emerged in 1997-1998 [3, 6]. However, there was diversity in the internal genes of the American viruses compared with Canadian isolates, mainly due to the presence of the TRIG cassette in the American isolates whereas the Canadian virus internal genes were classical swine or human in lineage.

The objective of the work described here was to study the pathogenesis and transmission of *δ*-cluster H1 influenza A viruses of swine in an experimental pig model, comparing three isolates from different locations of the USA (Texas, Minnesota, and Illinois), different collection times (2008, 2007, and 2005, resp.) and with full-length or truncated PB1-F2 accessory protein. PB1-F2 has been described as virulence factor for other influenza viruses in other species.

## 2. Materials and Methods

### 2.1. Virus Isolates

Three human-like *δ*-cluster H1 viruses (two from the *δ*2 sub-cluster and one from the *δ*1 sub-cluster) were used in the *in vivo* pathogenesis and transmission study. All viruses were isolated from postmortem lung tissue samples collected from pigs exhibiting clinical signs of respiratory disease and submitted to the University of Minnesota Veterinary Diagnostic Laboratory. The two *δ*2 sub-cluster H1N1 viruses, A/sw/Minnesota/07002083/07 H1N1 (MN07) and A/sw/Illinois/00685/05 H1N1 (IL05), were shown to have a predicted truncated PB1-F2 [6], whereas A/sw/Texas/01976/08 H1N2 (TX08) of the *δ*1 sub-cluster contained the full-length PB1-F2 coding sequence [5]. Putative viral protein sequences were highly conserved within the three viruses using published GenBank sequences for comparisons (IL05: FJ638301, FJ638299, FJ638297, FJ638295, FJ638302, FJ638300, FJ638298, FJ638296; MN07: FJ611902, FJ611900, FJ611898, FJ611896, FJ611901, FJ611899, FJ611897, FJ611895; TX08: CY082598, HM461847, HM461848, HM461849, HM461850, HM461851, HM461852, HM461853, HM461854). Identity at the amino acid level ranged from 94.8% to 99.0% with the exception of the NA genes (N1 compared with N2). The HA proteins had 98.4% identity within the *δ*2 sub-cluster viruses and 96.8% between the *δ*1 and *δ*2 sub-cluster viruses. Three additional *δ*-cluster isolates were used for heterologous hemagglutination inhibition (HI) assays, A/sw/North Carolina/00573/2005 H1N1 (NC05, *δ*2 sub-cluster), A/sw/Minnesota/02011/2008 H1N2 (MN08, *δ*1 sub-cluster), and A/sw/Illinois/07003243/07 H1N2 (IL07, *δ*1 sub-cluster). All isolates were propagated in MDCK cells following standard procedures.

### 2.2. *In Vivo* Study

Fifty 3-week-old cross-bred pigs were obtained from a herd free of influenza A virus and treated with ceftiofur crystalline-free acid (Pfizer Animal Health, New York, NY) to reduce bacterial contaminants prior to the start of the experiment. Pigs were housed in biosafety level 2 (BSL2) containment during the study and cared for in compliance with the Institutional Animal Care and Use Committee of the National Animal Disease Center. Pigs were divided into seven groups including three groups given primary inoculation (*n* = 10 per group) with each of the three *δ*-cluster viruses, three contact groups, and one uninfected control group. Primary pigs at four weeks of age were inoculated intratracheally with 2 × 10^5^ TCID_50_/mL of each virus (2 mL of inoculum), and naïve contact pigs (*n* = 5) were comingled with the primary inoculated pigs after 48 hours. All pigs were observed daily for clinical signs of disease and fever. Nasal swabs were taken on 0, 3, 5, 7, and 9 days post infection (dpi) or days post contact (dpc) to evaluate nasal shedding. Oral fluid (OF) samples were collected from each of the treatment groups using a cotton rope hung in each pen on days 3–10, 14, 18, and 20 pi as described previously [7]. The OF samples were assayed for influenza A viral RNA by a TaqMan real-time PCR assay for the influenza A matrix gene. In addition, viral RNA from pooled OF samples obtained at 3–9 dpi or 1–7 dpc was quantified.

Five pigs from each primary inoculated group were euthanized on day 5 pi to evaluate lung lesions and viral load in the lung. The remaining pigs (*n* = 5 per primary group and *n* = 5 per contact group) were euthanized at 21 dpi or 19 dpc. All pigs were humanely euthanized with a lethal dose of pentobarbital (Sleepaway, Fort Dodge Animal Health, Fort Dodge, IA). After euthanasia, lungs were aseptically removed at necropsy and lavaged with 50 mL MEM to obtain bronchoalveolar lavage fluid (BALF). Postmortem samples including serum, lung, and trachea were collected. Nonchallenged age-matched negative control pigs were necropsied on day 5 pi (*n* = 5 pigs).

### 2.3. Pathologic Examination of Lungs

At necropsy, lungs were removed and evaluated for typical lesions of influenza virus infection. The percentage of the surface affected with pneumonia was visually estimated for each lung lobe, and a total percentage for the entire lung was calculated based on weighted proportions of each lobe to the total lung volume [8]. Tissue samples from the trachea and right cardiac lung lobe and other affected lobes were taken and fixed in 10% buffered formalin for histopathologic examination. Lung sections were given a score from 0 to 3 to reflect the severity of bronchial epithelial injury using previously described methods [9].

### 2.4. RNA Extraction from OF Samples

Viral RNA from oral fluid samples was extracted using the MagMAx Viral RNA Isolation (Ambion) kit protocol with modifications. Briefly, clarified oral fluid (300 *μ*L) was added to the MagMax plate in duplicate and the volumes of the other reagents were increased proportionally. Isopropanol was omitted from the lysis/binding buffer initially and added after the oral fluid sample was mixed. RNA was quantified, and the TaqMan real-time PCR assay for the influenza A virus matrix gene was performed following protocol as previously described [10]. Mean quantities of influenza A virus RNA assessed by real-time RT-PCR of duplicate samples were transformed to log_10_ scale for comparison.

### 2.5. Viral Replication and Shedding

Tenfold serial dilutions in serum-free MEM supplemented with TPCK trypsin and antibiotics were made with each BALF sample and nasal swab filtrate sample. Each dilution was plated in triplicate in 100 *μ*L volumes onto PBS-washed confluent MDCK cells in 96-well plates. At 72 hours, plates were fixed and stained for immunocytochemistry [11]. A TCID_50_ titer was calculated for each sample [12].

### 2.6. Serologic Assays

The homologous and cross-HI assays were then performed with IL05, MN07, and TX08 as described previously [13]. In addition, 21 dpi and 19 dpc sera were evaluated against NC05, IL07, and MN08 in heterologous HI assays. Reciprocal HI titers were log_2 _transformed for analysis and reported as geometric means of the reciprocal titers (5 pigs per group).

### 2.7. Multicycle Growth Analysis

MDCK cells were infected in 24-well cell culture plate at low MOI (0.001) with the three *δ*-cluster H1 viruses. Supernatants were collected at 6, 12, 24, and 48 hours after infection and subsequently titrated on MDCK cells as described above. Each virus was tested in triplicate, and the results are representative of two independent experiments.

### 2.8. Statistical Analysis

Analysis of variance (ANOVA) with a *P*-value≤ 0.05 considered significant (GraphPad Prism, GraphPad Software, La Jolla, CA) was used to analyze log_10_ transformed BALF and nasal swab virus titers, log_2_ transformations of HI reciprocal titers, growth curve, and macroscopic or microscopic pneumonia scores. Response variables shown to have a significant effect by treatment group were subjected to pairwise comparisons using the Tukey-Kramer test.

## 3. Results

### 3.1. Clinical Disease, Macroscopic, Pneumonia and Microscopic Lung Lesion Scores

**Figure 1:**
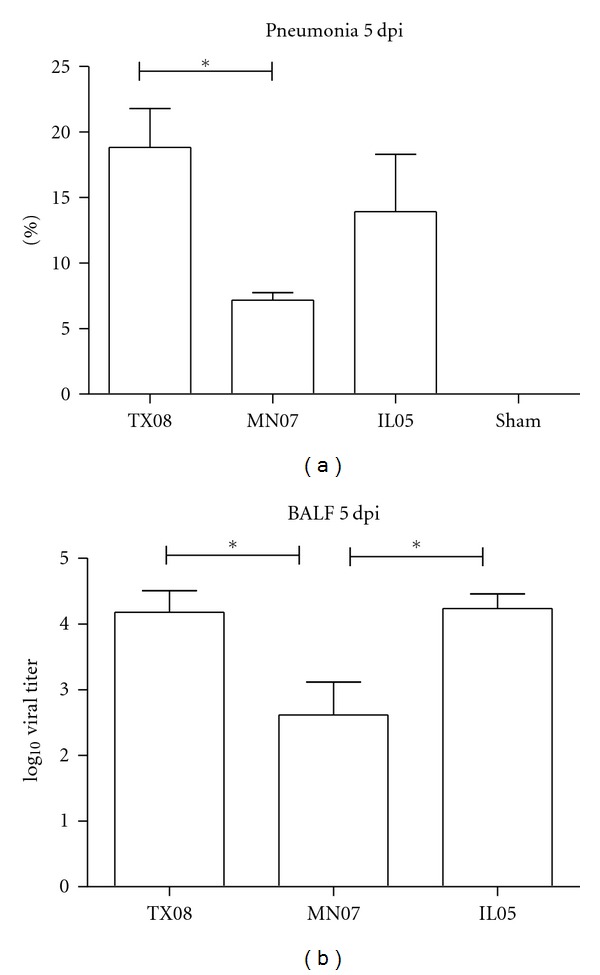
(a) Percentage of lung involvement at 5 dpi in pig groups inoculated with TX08, MN07, IL05 and sham. *Significantly different from TX08 versus MN07, TX08 versus sham, and IL05 versus sham at *P* < 0.05. (b) Virus titers in bronchoalveolar lavage fluid (BALF) at 5 dpi for primary inoculated groups. *Significantly different from TX08 versus MN07 and MN07 versus IL05 at *P* < 0.05.

All viruses induced typical influenza illness; however, clinical signs and macroscopic (Figure 1(a)) and microscopic lesions induced by MN07 were reduced in severity as compared to TX08-infected pigs. IL05-infected lung lesions versus sham showed significant differences at *P* < 0.05. At 5 days post infection (dpi), microscopic lesions in lungs and tracheas were also typical of influenza virus infection. Histopathologic lesions in lungs were characterized by necrotizing bronchiolitis with mild to moderate interstitial pneumonia. Significant differences in histopathological lesions in the trachea were not identified among groups. Negative control pigs remained negative for influenza A virus for the duration of the experiment.

### 3.2. Viral Replication and Transmission Efficiency

**Figure 2:**
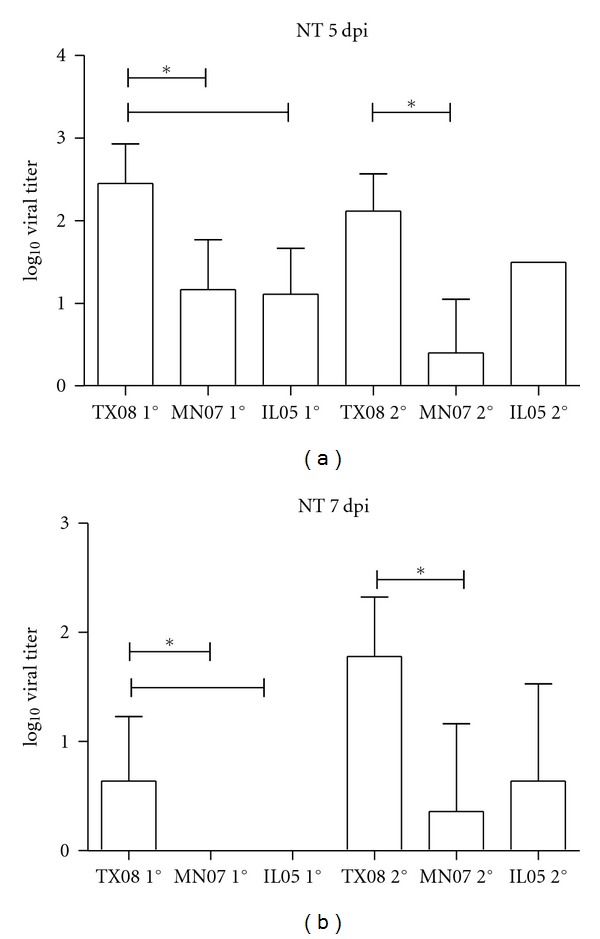
Quantity of virus detected in nasal swabs (NT) at 5 dpi (a) or 7 dpi (b) from pigs primarily inoculated (1°) with TX/08, MN/07, or IL/05 or in contact (2°) with inoculated pigs. *Significantly different from TX08 1° (primary) or TX08 2° (contact) versus MN07 1°, MN07 2°, or IL05 1° or IL05 2° and MN07 2° versus IL05 2° at *P* < 0.05. *Significantly different from TX08 2° versus MN07 1°, MN07 2°, or IL05 1° at *P* < 0.05.

**Figure 3:**
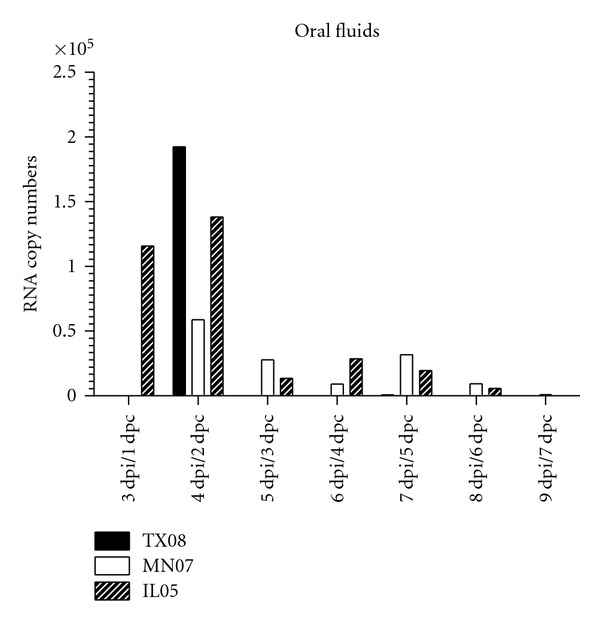
Quantitative influenza A virus real-time RT-PCR of RNA extracted from oral fluids. Mean quantity of duplicate samples was transformed to log_10_ scale.

MN07-infected pigs had lower virus titers in the lung at 5 dpi compared to TX08 and IL05 groups. Virus titers in BALF averaged 10^4.2^ TCID_50_/mL at 5 dpi in the TX08- and IL05-inoculated group and 10^2.6^ TCID_50_/mL at 5 dpi in the MN07-inoculated group (Figure 1(b)). All inoculated groups shed virus in nasal swab samples on days 3 and 5 pi. On day 3 pi, 95.5% of nasal swabs were positive with an average titer of 10^2.4^ TCID_50_/mL in pigs infected with TX08 and MN07 and 10^1.5^ TCID_50_/mL in pigs infected with IL05 (data not shown). On day 5 pi, 93% of nasal swabs were positive with an average titer of 10^2.4^ TCID_50_/mL for pigs infected with TX08, 10^1.2^ TCID_50_/mL for pigs infected with MN07, and 10^1.1^ TCID_50_/mL for pigs infected with IL05 (Figure 2(a)). Pigs in contact with TX08-infected pigs also shed significantly more virus in nasal secretions at 5 days post contact (dpc, Figure 2(a)). On day 7 pi, all nasal swab samples from MN07- and IL05-inoculated pigs were negative. In contrast, 80% of the pigs in the TX08-inoculated group continued to shed virus in nasal secretions on day 7 pi, with an average titer of 10^0.7^TCID_50_/mL (Figure 2(b)). In addition, nasal secretions from TX08 virus contact pigs had higher mean virus titers at 7 dpc. In the TX08 contact group, 100% of the pigs had positive nasal swab samples on days 3, 5, and 7 pc, with average titers of 10^2.7^, 10^2.1^, and 10^1.8^ TCID_50_/mL, respectively. Far fewer contact pigs were shedding virus in the MN07 and IL05 groups, with just 20% and 40% nasal swab positive, respectively, at 7 dpc. These data demonstrate that all viruses were able to replicate in the lung and nasal mucosa; however, nasal shedding of TX08 was greater in magnitude and duration. In contrast to nasal shedding, MN07 and IL05 viral RNA was detected more frequently in OF in comparison to TX08 (Figure 3). The virus shedding and detection kinetics in OF were different when compared to nasal swabs.

**Figure 4:**
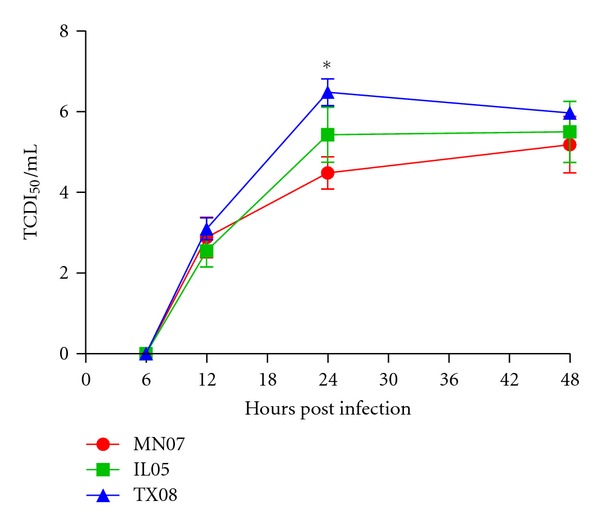
Multistep growth curves of TX08, MN07, and IL05 in MDCK cells. Confluent MDCK cells were infected at an MOI of 0.001 TCDI_50_/mL. Virus yields (log_10_ TCDI_50_/mL) at 6, 12, 24, and 48 h post infection were titrated in MDCK cells. The results are representative of two independent experiments. Each virus was treated in triplicate in both experiments. TX08 grew at statistically significant higher titers with respect MN07 at 24 hours post infection (*P* < 0.05). *Significantly different.

To further evaluate the replicative ability of the *δ* virus isolates, we studied the growth of MN07, TX08, and IL05 in MDCK cells in multicycle growth curve analyses. The three viruses grew similarly at each time point although viral yields were significantly different (*P* < 0.05) between TX08 and MN07 at 24 hours post infection (Figure 4).

### 3.3. Humoral Immune Response and Cross-Reactivity

Pigs in this study mounted a more robust humoral immune response to MN07 and IL-05 *δ*2 cluster H1N1 viruses compared to the TX08 in homologous HI assays (Table 1). Cross-HI using heterologous virus demonstrated little cross-reactivity between the *δ*1 TX08 antisera and the *δ*2 IL05 and MN07 viruses (Table 2). As expected for viruses within the same sub-cluster and sharing higher similarity, there was greater cross-reactivity between the MN07 virus and IL05 antisera. Heterologous HI tests using additional H1 *δ*-cluster influenza A viruses from swine were conducted to evaluate cross-reactivity of 21 dpi serum samples from primary infected pigs (Table 2). The *δ*1 sub-cluster was represented by IL07, TX08, and MN08, and the *δ*2 sub-cluster was represented by IL05, NC05, and MN07 viruses (Table 2). As expected, cross-reactivity was found within sub-clusters, specifically between TX08 antisera and IL07 and between IL05 and MN07 antisera against IL05, MN07, and NC05 viruses. Results showed little cross-reactivity between MN08 and any of the antisera. The greatest cross-reactivity was demonstrated between IL07 and TX08 antisera generated against the H1 *δ*1 sub-cluster. Antisera from pigs infected with IL05 had significant cross-reactivity against NC05, IL07, and MN07 spanning some but not all of the representative viruses in the *δ* sub-clusters. In general, the antigenic cross-reactivity is consistent with phylogenetic evidence of 2 sub-clusters of *δ*-H1 influenza A viruses of swine, although the antigenic variability appears to be complex.

## 4. Discussion

Classical H1N1 influenza A viruses were the main cause of swine influenza in North America until 1998. Since the emergence of viruses possessing the TRIG cassette, an increase in the rate of genetic change in North American swine influenza isolates appears to have occurred in H1 virus subtypes, and distinct genetic and antigenic clusters have evolved [13]. The introduction of human origin surface glycoproteins in the TRIG backbone further complicated the swine influenza epidemiology in North America. Human-like H1 influenza viruses were first identified in pigs in Canada in 2003-2004 [4]. In the USA, human-like isolates began to be identified in swine in 2005 [6]. Full-genome sequencing characterized these as triple reassortants with the TRIG constellation similar to that of the contemporary triple reassortant H3N2, H1N1, and H1N2 viruses in North American swine, but with HA and NA most similar to human seasonal H1 influenza virus lineages [6]. Until recently, isolation of human-lineage H1 viruses from swine has been rare in comparison with classical swine-lineage and avian-lineage viruses. However, epidemiological data from 2009 and 2010 years suggest that the human-lineage H1 viruses have become one of the most frequently detected genotype in swine herds in the USA [14]. Therefore, a comparative experimental study in pigs of isolates from different phylogenetic sub-clusters, year of isolation, and geographic region was necessary since little is described about the biological properties of this newly emerged group of swine viruses.

Our study indicates that the three isolates of *δ*-cluster H1 influenza A viruses evaluated can efficiently replicate, transmit, cause lung pathology, and induce humoral immune responses in a experimental study in the swine host. However, the kinetics of virus replication, pathogenesis, as well as host humoral immune response differed among the three viruses, with TX08 being more virulent. A previous study from our group evaluated the pathogenicity and transmission properties of the human-like *δ*-cluster H1N1 MN07 (A/sw/MN/07002083/07) in comparison to a 2004 reassortant H1N1 (A/sw/IA/00239/04) with swine lineage HA and NA [6]. The MN07 isolate induced disease typical of influenza virus and was transmitted to contact pigs; however, it replicated less extensively than the 2004 H1N1 [6].

Herein, we compared three *δ*-cluster H1 influenza A virus isolates in pigs, including the MN07 isolate described in the previous study. Our report is the first study comparing the pathogenesis and transmission of both sub-clusters of the *δ*-cluster H1 influenza viruses in swine. All isolates induced disease typical of influenza virus and were transmitted to contact pigs, as demonstrated by nasal swab virus isolation and seroconversion. However, clinical signs as well as macroscopic lung lesions induced by MN07 were less severe than in TX08-infected pigs. MN07-infected pigs had lower virus titers in the lung than the TX08 and IL05 groups as demonstrated by virus titers in BALF at 5 dpi. TX08 virus was more efficient in nasal shedding and transmission to contact pigs. Additionally, all contact pigs were seropositive by 12 dpc, with reciprocal HI titers ranging from 40 to 160. Comparison of replication kinetics in the upper respiratory tract via nasal secretions also showed differences among the three isolates. All viruses were able to replicate and transmit; however, TX08 nasal shedding in contact pigs was at higher titers in a greater percentage with an extended duration. Multistep growth analysis in MDCK cells revealed that TX08 had higher titers at 24 hours post infection with respect to IL05 and MN07.

Quantitative analysis of viral RNA in oral fluids showed different kinetics of detection in comparison with nasal swabs as TX08 samples presented higher viral RNA levels in oral fluids but were of shorter duration when compared with MN07 and IL05. The higher quantity, but shorter duration, of detection of influenza RNA from OF in the TX08 group is likely explained by the severity of clinical signs in pigs inoculated with this isolate. Clinically affected, lethargic pigs are less likely to chew on the cotton fiber rope used to collect the oral fluids.

Humoral responses to IL05 and TX08 were inversely related to virus titers in nasal swabs. Pigs in this study mounted a more robust humoral response by HI titers to MN07 and IL-05 *δ*2-cluster H1N1 compared to the TX08 H1N2. Cross-HI using heterologous virus demonstrated little cross-reactivity between antisera and viruses from different H1 *δ* sub-clusters. Based on phylogenetic and antigenic analysis, two sub-clusters of the *δ*-cluster H1 influenza A viruses appear to have emerged in swine within a period of 2 years with limited serologic cross-reactivity between them. The viruses adopted in this study show amino acid differences in all putative encoded proteins (data not shown). None of these differences involve residues that have been previously shown to modulate either pathogenicity or transmission in pigs. Noteworthy, TX08 shows 187 N (H1 numbering) in the HA protein compared to 187D of MN07 and IL05. D187N is a substitution often acquired by the human seasonal H1. It has been recently shown that a reverse-genetics recombinant human seasonal virus containing D187N retained the binding activity to the *α*2-6 linked sialosides recognized by the 187D virus; however, it acquired the capability to bind a wide range of *α*2-3-linked sialosides [15, 16]. Since pigs express both *α*2-6 and *α*2-3 receptors, D187N may have played a role in the pathogenesis of TX08, possibly through increased receptor binding activity in the lower respiratory tract, where *α*2-3 receptors have been shown to colocalize with *α*2-6 receptors [17, 18].

Based on sequence prediction, TX08 should express the full-length PB1-F2 accessory protein whereas the remaining two viruses have premature truncations encoded in the gene. How the presence of full-length PB1-F2 can influence the pathogenesis of swine influenza viruses in the swine host is still unclear and further experiments are warranted.

## 5. Conclusions

The findings of this study demonstrate the diversity and continuous evolution of influenza A viruses of swine, as well as varying pathogenicity and ability to replicate and transmit to contact pigs. Not only are these findings important for swine health, but they may have implications for human health as well. Since the seasonal H1N1 component of the trivalent human vaccine was replaced by the 2009 pandemic H1N1 virus, the human population immunity to seasonal H1N1 may begin to wane. As a result of waning immunity, the human population may become more susceptible to variant H1 viruses that have adapted and evolved in other hosts. Therefore, it will be necessary to monitor the evolution of the *δ*-cluster viruses in the swine population as they may become a possible reservoir for spillover back into the human population. Furthermore, determination of virulence, adaptation, and transmission factors of influenza virus in the swine host are critical for risk assessment of viruses emerging the swine host as well as risk to humans.

## Figures and Tables

**Table 1:** Results of homologous HI assays using *δ*-cluster H1 influenza A viruses TX08, MN07, and IL05.

Serum	Seroconversion	
	12 dpc/14 dpi	19 dpc/21 dpi
TX08 H1N2		
Primary	5/5 (61)	5/5 (106)
Contact	5/5 (70)	5/5 (80)
MN07 H1N1		
Primary	5/5 (211)	5/5 (211)
Contact	5/5 (279)	5/5 (368)
IL05 H1N1		
Primary	5/5 (211)	5/5 (211)
Contact	5/5 (106)	5/5 (106)

Number positive with reciprocal HI ≥ 40/total tested (geometric mean reciprocal titer against homologous antigen).

**Table 2:** Geometric mean reciprocal titers from cross-HI assays using *δ*1 or *δ*2 H1 influenza viruses of 21 dpi serum from primary inoculated pigs. Viruses are listed in the far left column and antisera are listed in the top row. Homologous titers are in bold.

		TX08	IL05	MN07
		*δ*1 H1N2	*δ*2 H1N1	*δ*2 H1N1
*δ*1	IL07	106	70	61
	TX08	**106**	<10	<10
	MN08	26	<10	<10
*δ*2	IL05	53	**211**	53
	NC05	80	160	61
	MN07	40	160	**211**
